# Impending Chemotherapeutic Impact of *Arthrospira platensis* Nanoparticles and/or Sorafenib against Hepatocellular Carcinoma through Modulation of Antioxidant Status, Tumor Marker Genes, and Anti-Inflammatory Signaling Pathways

**DOI:** 10.3390/toxics11020107

**Published:** 2023-01-22

**Authors:** Heba I. Ghamry

**Affiliations:** Department of Home Economics, College of Home Economics, King Khalid University, P.O. Box 960, Abha 61421, Saudi Arabia; hgmry@kku.edu.sa

**Keywords:** DENA, *Arthrospira platensis*, sorafenib, cytokines, PPAR-γ, FOXO-1

## Abstract

This study investigated *Arthrospira platensis* nanoparticles (NSP) to overcome sorafenib resistance in diethyl nitrosamine-induced hepatocellular carcinoma (HCC) in rats. This study used sixty Wistar male rats randomly grouped into two main groups, the normal control group, and the HCC model. For the normal control group (*n* = 12), animals were injected i.p. with PBS two times/week for 16 weeks. The remaining 48 rats were injected i.p. with using a single dose of diethyl nitrosamine (DENA) (200 mg/kg, ip), followed by phenobarbital sodium (0.05%) in drinking water for 16 weeks. At the end of the 16th week, rats were allocated into four groups (11 rats/each), one group was left without treatment (DENA group), and the other three groups were treated with either sorafenib (30 mg/kg; p.o.) or *Arthrospira platensis* Nanoparticles (NSP) (0.5 mg/kg body weight) once daily orally with the aid of gastric gavage or their combination for another four weeks. Blood and tissue samples were collected for further biochemical, histological, immunohistochemical, and gene expression analysis. Our result revealed that DENA-treated rats showed a marked elevation of hepatic enzyme markers with an increase in the total protein and globulin and decreases in the hepatic SOD. Catalase and GSH, with significantly increased MDA levels, subsequently increased the tumor biomarkers (AFP and CEA). On the molecular level, the DENA-treated rats showed significant up-regulation of Cyp19 mRNA and the inflammatory cytokines (TNF-α, iNOS, and TGF-1β) as well as the Ki-67 gene expression (*p* < 0.05) with down-regulation of the PPAR-γ and FOXO-1. In addition, the HCC group showed a loss of hepatic architecture, as well as atypia, swelling, macrosteatosis of hepatocytes, and fibrosis, besides increased vascularization. The immunohistochemical findings show increased expression of both GPC-3 and Hep Par 1 in the HCC group. SOR, NSP, or a combination of NSP and SOR.NSP treatment significantly overturned the DENA’s harmful effect near the normal levels and restored all cancer biomarkers and antioxidant activities, indicating the chemotherapeutic impact of NSP. The present study provides evidence that NSP exerts a major anticancer effect on DENA-induced HCC. SOR/NSP is a promising combination for tumor suppression and overcoming sorafenib resistance in HCC by modulating antioxidants, anti-inflammatory signals, and tumor markers.

## 1. Introduction

Primary liver cancer is the second leading cause of cancer death in men and the third in both sexes [1]. Hepatocellular carcinoma causes 75% of liver cancers (HCC). Resection, ablation, and transplantation cure HCC. These surgeries require a fast diagnosis [2]. Molecular changes at the genetic and epigenetic levels can both produce hepatocarcinogenesis [3]. Dysfunction in caspases and the p53 pathway, as well as an imbalance between pro- and anti-apoptotic Bcl2 family members, are major regulators of apoptosis [4]. Anticancer drugs could potentially target crosstalk between these pathways. Sorafenib (SOR) is a multi-kinase inhibitor that inhibits several serine/threonine kinases [5]. Despite its limitations, it is the only anti-angiogenic medicine recommended for treating advanced HCC by the American Association for the Study of Liver Diseases (AASLD) [6].

Sorafenib inhibits tumor formation and proliferation by blocking key tyrosine kinase pathways like VEGFR, EGFR, and PDGFR [7]. Around 40% of HCC patients benefit from SOR due to genetic variability and other factors [7]. The development of resistance to sorafenib compounds is a major problem in its administration [8]. Cell proliferation, metastasis, autophagy, and acquired resistance to SOR are all greatly aided by the activation of biochemical cascades such as PI3K/AKT/mTOR and NF-κB [9].

Antioxidants protect cells from oxidative damage and reduce the production of free radical species; their use is becoming increasingly popular [10]. Spirulina is a type of filamentous cyanobacterium (fresh blue–green algae) called *Arthrospira platensis,* rich in Phyto components like protein-building amino acids and lipids. It is a good source of riboflavin, vitamin B, carotenoids, and other pigments [11]. *Aspergillus* is a major contributor to fungal secondary metabolites that provide numerous biological benefits [12]. *A. awamori* supplementation has decreased lipid peroxidation in skeletal muscle, increased development efficiency, and acted as an anticancer and antioxidant [13,14]. High quantities of coumarins, glucose, saponins, flavonoids, and tannins with antioxidant action were discovered in *A. awamori* [15]. Because of their small size and active surface, Nanoparticles are special active chemicals. Nanoparticles have greater and more sustained activity than conventional particles because they can circulate in the bloodstream for longer [16]. Thus, the current work studies the effect of *A. platensis* nanoparticles on SOR resistance and the intracellular pathway by which they may be beneficial in reducing infertility caused by diabetes. This study aimed to assess the efficacy of SOR/*A. platensis* nanoparticles used, either singly or in combination, in treating HCC and overcoming treatment resistance in HCC.

## 2. Materials and Methods

### 2.1. Chemicals

Diethylnitrosamine (DEN) was purchased from (DEN, Sigma-Aldrich Co., St. Louis, MO, USA) and was dissolved in NaCl 0.9% (1 g in 25 ml). Sorafenib was obtained from Bayer (Berlin, Germany). Nanoparticles of *Arthrospira platensis* (NSP) were prepared following a previous method [17], with a size particle of 158 nm. See the supplementary materials Figures S1–S4.

Serum alpha-fetoprotein (AFP) concentrations were estimated using kits obtained from Kamiya Biomedical Company, Washington, USA. Serum biochemical parameters were measured using ready-made kits to determine alanine aminotransferase (ALT), aspartate aminotransferase (AST), alkaline phosphate (ALP), and gamma-glutamyltransferase (GGT) obtained from (Randox Laboratories Ltd., Crumlin, County Antrim, UK). Ready-made kits for reduced glutathione (GSH), superoxide dismutase (SOD), and malondialdehyde (MDA) were purchased from Biodiagnostics, Cairo, Egypt. p53 antibodies were obtained from Biotechnologies, (Santa Cruz Biotechnology, Santa Cruz, CA, USA).

### 2.2. Animals

This study used sixty Wistar male rats (6–8 weeks of age, weighing 165–185 g). Animals were housed in polycarbonate cages in a room free from any source of chemical contamination, and water and food were given ad libitum for 12 h darkness: 12 h light with a controlled temperature (25 °C) and humidity (53%). Rats obtained from the Experimental Animal Care Centre, College of Medicine of King Khalid University, Abha, Saudi Arabia. Rats were adapted for 15 days before the experiments. The rats were cared for following the guidelines established by the NIH for appropriately handling and managing animals. The ethics board of King Khaled University in Saudi Arabia approved all procedures. The ethical approval was given the number ECM#2020–1801.

### 2.3. Experimental Design

After acclimatization, rats were randomly grouped into two main groups, the normal control group and the HCC model. For the normal control group (*n* = 12), animals were injected i.p. with PBS two times/week for 16 weeks. The remaining 48 rats were injected i.p. with using a single dose of diethylnitrosamine (DENA) (200 mg/kg, ip), followed by phenobarbital sodium (0.05%) in drinking water for 16 weeks [18]. Induction of HCC was confirmed by histopathological analysis. At the end of the 16th week, rats were allocated into four groups (11 rats/each); one group was left without treatment (DENA group), and the other three groups were treated with either sorafenib (30 mg/kg; p.o.) dissolved in DMSO (Sigma, St. Louis, MO, USA) [19], *Arthrospira platensis* Nanoparticles (NSP) (0.5 mg/kg body weight) were dissolved in normal saline [17], once daily orally by the aid of gastric gavage or their combination for further 4 weeks.

Blood samples were collected via retro-orbital Venus plexus bleeding under light ether anesthesia. Blood samples were centrifuged at 1008 g for 15 minutes. Sera were kept at −80 °C for further biochemical analysis. 

The entire liver was examined and weighed. Rats (*n* = 8) were chosen randomly from each group for necropsy after an intravenous injection of sodium pentobarbital at a dose of 30 mg/kg to avoid suffering. The liver was removed from each rat and 200–300 mg was manually homogenized in cold phosphate buffer saline (PBS). The homogenate was centrifuged at 5000 g for 15 min at four °C, and the supernatant was kept at −20 °C. Another part of the liver was formalin-fixed at 10% for histological investigation.

### 2.4. Biochemical Parameters

Total protein (TP), globulin, albumin, and the aminotransferases alanine aminotransferase (ALT), aspartate aminotransferase (AST), alkaline phosphatase (ALP), and gamma-glutamyl transferase (GGT) were measured using commercial kits from Bio-diagnostic Co. (Giza, Egypt). Serum-fetoprotein (AFP, LS-F22287) and carcinoembryonic antigen (CEA, LS-F5892) were measured using enzyme-linked immunosorbent assays (ELISA) (Lifespan Biosciences, Inc., Seattle, WA, USA).

### 2.5. Antioxidant Status Markers in Liver

A reduced glutathione concentration was assessed(CAT. No. GR 25 11) [20]. Malondialdehyde (MDA), a major byproduct of lipid peroxidation, was measured as an indicator of lipid oxidation following the manufacturer’s instructions (Bio-diagnostic Co., Giza, Egypt. CAT. No. MD 25 29) [21]. Superoxide dismutase (SOD) activity was measured according to [22], depending on the ability of the enzyme to hinder the phenazine methosulphate-mediated reduction of nitroblue tetrazolium (NBT) dye, photometrically measured at 560 nm. Catalase (CAT) was used according to the manufacturers’ instructions (Bio-diagnostic Co., Giza, Egypt. CAT. No. CA 25 17 and CAT. No. SD 25 21 for CAT and SOD, respectively).

### 2.6. Gene Expression-Analysis (RT-PCR)

We used RT-PCR to examine hepatic gene expression. Total RNA was isolated from 100 mg of liver tissue using TRIzol (Invitrogen, Life Technologies, Carlsbad, CA, USA). TRIzol was used to extract total RNA from 100 mg of liver tissue, and a Nanodrop was used for quantification (Uv–Vis spectrophotometer Q5000/Quawell, San Jose, CA, USA). DNA was synthesized from RNA samples having an A260/A280 ratio of 1.8 or above using a cDNA synthesis kit (Fermentas, Waltham, MA, USA). SYBR Green master mix (Roche, Basel, Switzerland) and the primers listed in Table 1 were used for cDNA amplification. The conditions of quantitative RT-PCR amplification commenced with initial reverse transcription for the cDNA synthesis for 10 min at 55 °C. The resulting cDNA underwent 40 cycles of PCR with denaturation at 95 degrees Celsius for 5 s, annealing at 55 to 58 degrees Celsius for 25 seconds, and extension at 72 degrees Celsius for 15 seconds using the common housekeeping gene glyceraldehyde-3-phosphate dehydrogenase (GAPDH). Rotor-Gene Q (Qiagen, Valencia, CA, USA) was used to gather data and assess the threshold cycle value (Ct) mechanically. 2−ΔΔ methods were applied to the analysis of amplification data [23].

### 2.7. Histopathological and Immunohistochemical Examination

The histopathological and immunohistochemical examination was adopted according to [24]. Briefly, liver samples were taken immediately after dissection and fixed in a 10% neutral buffer formaldehyde solution for 24 hours. The fixed samples were dehydrated using ascending concentrations of ethyl alcohol (70% to absolute alcohol), then cleared in xylene and impregnated in paraffin wax. Tissue sections of 4–5 µm thickness were cut by a rotary microtome (Leica RM 2035, Leica Biosystems, Nusloch, Germany) and stained with hematoxylin and eosin, according to Bancroft and Gamble (2007). The tissue was examined and imaged using a light microscope (Olympus CX 40, Melbourne, Australia). The severity of pathological lesions was assessed according to the percentage of tissue affected in the entire section [25].

The immunohistochemical staining was carried out following a previous study [26], in which the paraffin sections were adhered onto coated slides, cleared in xylene, rehydrated, and then immersed in antigen retrieval (EDTA solution, pH 8). To remove nonspecific staining, the slides were incubated in 0.3% H_2_O_2_ and blocked in 5% bovine serum albumin in Tris-buffered saline (TBS) for two h. The liver slides were stained with Glypican-3 (GPC-3) mouse monoclonal immunoglobulin 12 (Biomosaics, Burlington, VT, USA), Hepatocyte Paraffin 1 (Hep Par 1) monoclonal mouse anti-human hepatocytes, and Clone OCH1 (Dako, Denmark) then washed with PBS for three consecutive times (20 min each), incubated with biotinylated secondary antibodies for 30 min, followed by avidin–biotin–peroxidase complex for another 30 min according to the instructions of the manufacturer (Universal Detection Kit, Dako, Denmark). Finally, the immune reaction was visualized as a brown color with 3, 3-diaminobenzidine tetrahydrochloride (DAB, Dako K0114 Kit) for 5 min, then washed in distilled water. Then, the slides were counterstained with Mayer’s hematoxylin for 1 min before mounting. Two pathologists reviewed the histopathological characteristics of each specimen to confirm the diagnosis, and the labeling index of GPC-3 and Hep Par 1 is expressed as the percentage of positive cells per total 1000 counted cells using eight high-power fields.

### 2.8. Statistical Analysis

GraphPad Prism 5 was used for all statistical analysis (GraphPad Software, San Diego, CA, USA). Data are described as mean ± SEM. A one-way analysis of variance followed by Tukey’s post hoc multiple range tests was used to examine the data. The level of significance was set at *p* < 0.05.

## 3. Results

### 3.1. Body Weight and Liver Weight

Rats given DEN had significantly lower (*p* < 0.05) final body weight, weight gain, with significant improvements in the HCC-treated groups with a combination of NSP and SOR (Table 2). In addition, a significant increase in liver-to-body weight ratio was noted in the HCC group concerning sthe other treated and control groups. 

### 3.2. Hepatic and Oxidative Injury Markers

**Figure 1:**
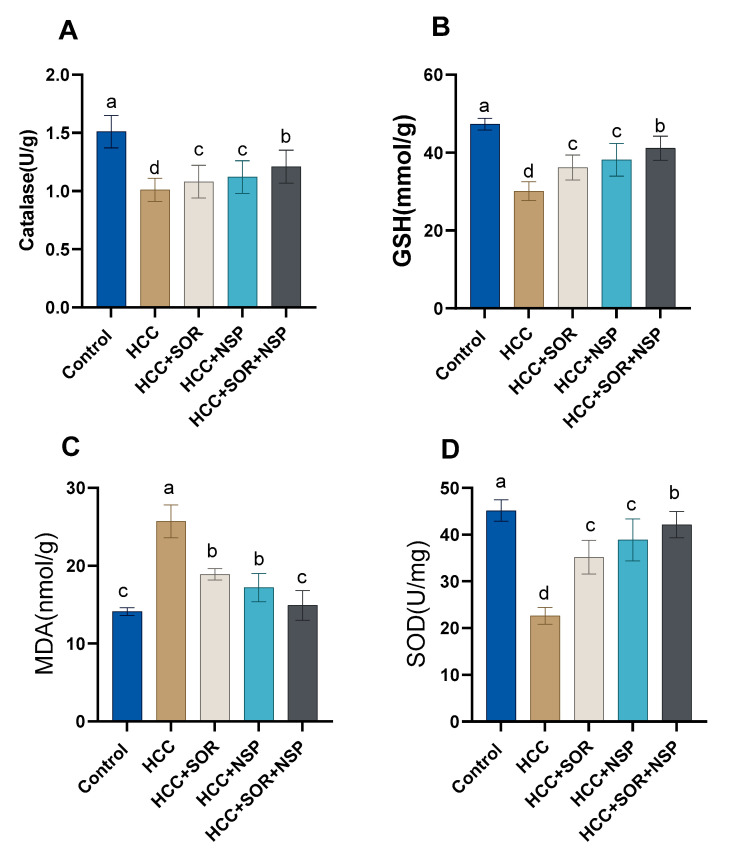
Effects of SOR, NSP, and their combination in DENA-induced HCC on the antioxidant status. (**A**) Catalase; (**B**) GSH; (**C**) MDA; (**D**) SOD. Data are presented as the mean ± SE. ^a, b, c, d^ Mean values with different letters differ significantly at (*p* ≤ 0.05). (*n* = 8).

**Figure 2:**
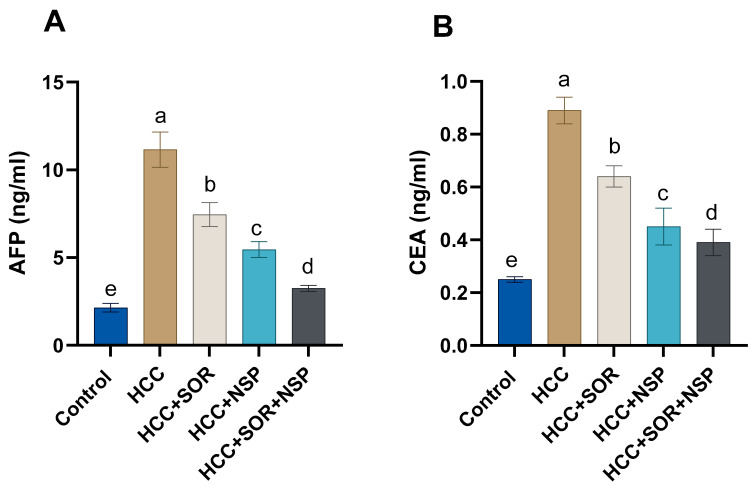
Effects of SOR, NSP, and their combination in DENA-induced HCC on the antioxidant status. (**A**) AFP; (**B**) CEA. Data are presented as the mean ± SE. ^a, b, c, d^ Mean values with different letters differ significantly at (*p* ≤0.05). (*n* = 8).

Oral administration of NSP and SOR and their combinations substantially overturned the negative impact caused by DEN, as shown in Table 3. The DEN-treated rats had increased ALT, AST, GGT, and ALP, and significant increases in total blood protein and globulin were also seen in the DENA-treated group. However, decreases in albumin and the A/G ratio were seen. The hepatic catalases and GSH levels were significantly decreased in the DEN-treated rats (*p* < 0.01), with significant increases in the hepatic MDA levels compared to the other treated and control groups. Inside out, significant increases (*p* < 0.05) in the HCC-treated groups with SOR and or NSP or in addition, the NSP in combination with SOR showed significant increases in the activity of the GSH, SOD, and CAT concerning the SOR- or NSP-treated rats as shown in Figure 1. Figure 2 outlines the tumor biomarkers (AFP and CEA). Tumor biomarker levels in DENA-treated animals increased altogether (*p* < 0.05) concerning the control group. Surprisingly, AFP and CEA levels were considerably (*p* < 0.05) reduced by the administration of SOR or NSPI alone or in combination relative to DEN-treated rats.

### 3.3. Molecular Analysis

**Figure 3:**
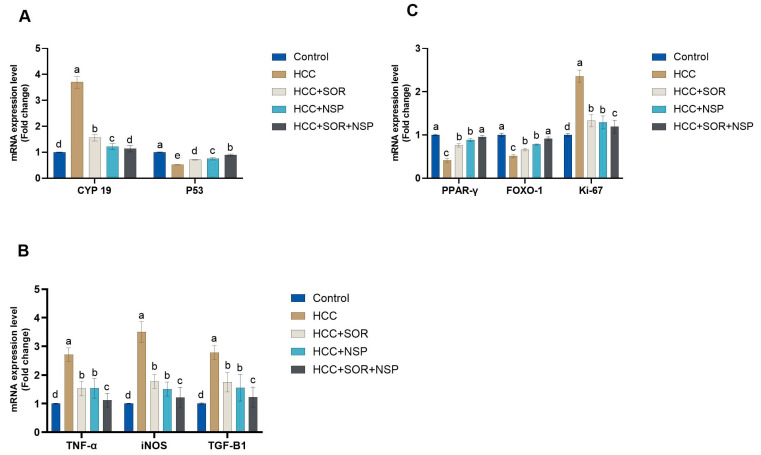
Effects of SOR, NSP, and their combination in DENA-induced HCC on the antioxidant status. (**A**) AFP; (**B**) CEA. Data are presented as the mean ± SE. ^a, b, c, d^ Mean values with different letters differ significantly at (*p ≤* 0.05). (*n* = 8).

Gene expression analysis revealed a marked upregulation of Cyp19 mRNA (*p* < 0.01) in the DEN-treated rats compared to the other treated groups. In contrast, the treatment of HCC rats with SOR, NSP, or combined NSP/SOR resulted in a significant downregulation (*p* < 0.05). Nevertheless, the expression level of p53 mRNA was significantly decreased (*p* < 0.01) in the DEN-treated rats as compared with the other treated and control groups. In contrast, the treatment of HCC rats with SOR, NSP, or combined NSP /SOR resulted in a significant upregulation (*p* < 0.05), as shown in Figure 3.

The anti-tumor activity of the NSP was confirmed by investigating its effect on the expression of inflammatory cytokines (TNF-α, iNOS, and TGF-1β). This current study has shown that the expression level of these inflammatory cytokines was significantly up-regulated (*p* < 0.05) in the HCC-induced group relative to those in the control and DEN-treated groups, as well as relative to rats treated with SOR, NSP, or combined NSP /SOR.

In addition, Figure 3 shows that the Ki-67 gene expression in the DENA group was up-regulated (*p* < 0.05) compared to the other treated and control groups. Ki-67 mRNA expression in the liver tissue of HCC rats treated with either SOR, NSP, or a combination of NSP and SOR was significantly downregulated (*p* < 0.05) compared to the DENA group.

Additionally, the current results showed a substantial (*p* < 0.05) down-regulation of the PPAR-γ and FOXO-1 mRNA expressions in the DENA group concerning the other treated and control groups. On the other hand, treatment with SOR, NSP, or a combination of NSP and SOR resulted in a significant (*p* < 0.05) up-regulation of the PPAR-γ and FOXO-1 genes expressions in hepatic liver tissue concerning the DENA-treated group

### 3.4. Histopathological and Immunohistochemical Findings

**Figure 4:**
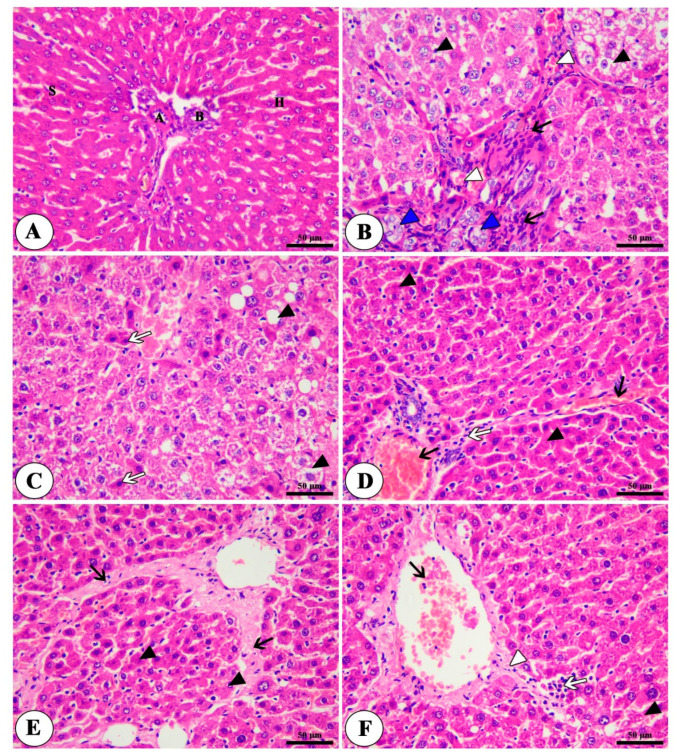
Photomicrograph of Liver tissue of different treated groups; control group (**A**) showing normal architecture of liver, hepatic artery (**A**), bile duct (**B**), polyhedral-shaped hepatocytes (H) arranged in a cord-like pattern and separated by blood sinusoids (S). HCC group (**B**,**C**) showing loss of hepatic architecture, cytologic atypia (blue arrow heads), swelling and macrosteatosis of some hepatocytes (black arrowhead), the presence of cells with strong acidophilic cytoplasm and hyperchromatic nuclei (white arrows), new blood vessel formation (white arrow heads) and fibrosis (black arrows). NSP-treated group (**D**) showing a moderate atypia of hepatocytes (black arrow heads), congestion of hepatic blood vessels (black arrows) and mild mononuclear cell infiltration (white arrows). Sorafenib-treated group (**E**) showing moderate atypia and degenerative changes of hepatocytes (black arrow heads), fibrosis (black arrows). NSP + sorafenib-treated group (**F**) showing normal hepatic architecture with mild hepatocellular degeneration (black arrow head), mild fibrosis (white arrow head), mild congestion of the central vein (black arrow) and mild mononuclear cell infiltration (white arrows). H&E, Bar = 50 µm.

The hepatic tissue of the control group revealed the presence of intact polyhedral cells, hepatocytes, with centrally located round nuclei with prominent nucleoli arranged in a cord-like pattern separated from each other’s by hepatic sinusoids (Figure 4A). The liver tissues of the HCC group showed a loss of hepatic architecture, atypia, swelling, macrosteatosis of hepatocytes and fibrosis, in addition to increased vascularization (Figure 4B,C). The HCC group treated with NSP showed mild atypia of hepatocytes with severe congestion of hepatic blood vessels, mild fibrosis and mononuclear cell infiltration (Figure 4D) while the group treated with sorafenib revealed mild atypia in hepatocytes in addition to marked fibrosis (Figure 4E). The group treated with both NSP and sorafenib showed a marked ameliorative effect represented in the normal hepatic architecture, with a mild congestion of hepatocytes and sinusoids, mild degeneration of hepatocytes with mild mononuclear cell infiltration, and mild fibrosis (Figure 4F and Table 4).

**Figure 5:**
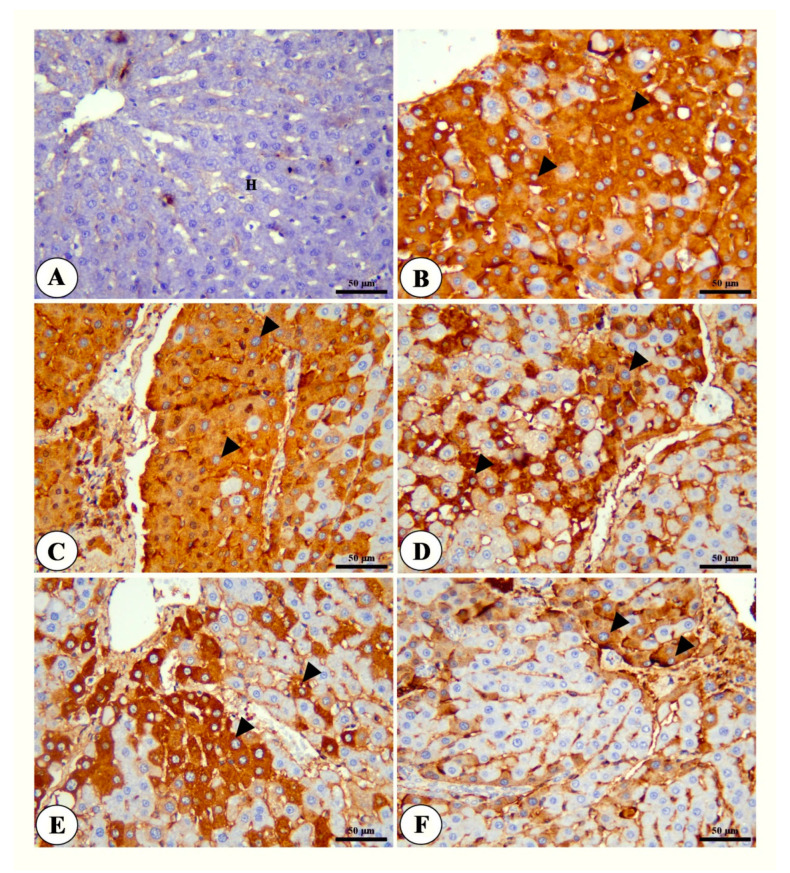
Photomicrograph of liver tissue of control group (**A**), HCC group (**B**,**C**), NSP-treated group (**D**), sorafenib-treated group (**E**) and NSP + sorafenib-treated group (**F**) showing positive cytoplasmic immunostaining of GPC-3 (black arrow heads). GPC-3 IHC, Bar = 50 µm.

**Figure 6:**
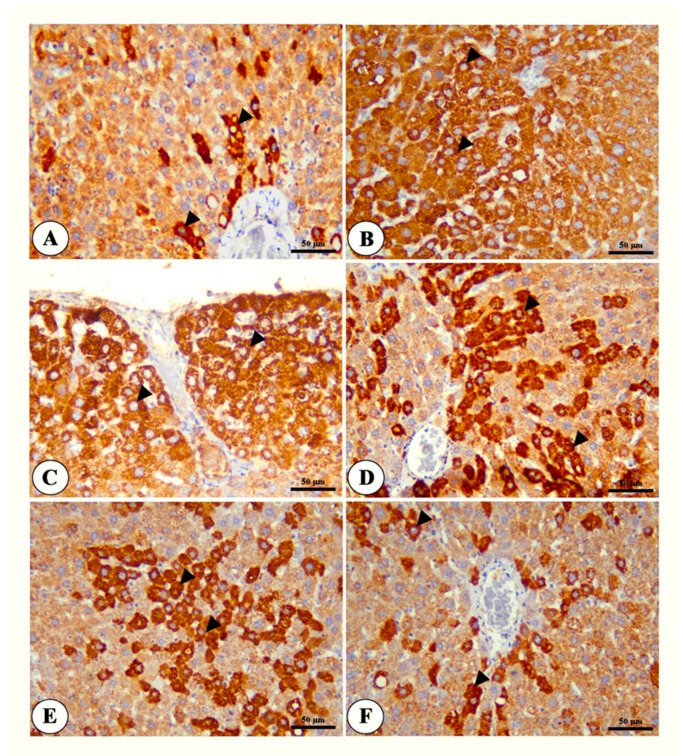
Photomicrograph of liver tissue of control group (**A**), HCC group (**B**,**C**), NSP = treated group (**D**), sorafenib-treated group (**E**) and NSP + sorafenib-treated group (**F**) showing positive cytoplasmic immunostaining of Hep Par 1 (black arrow heads). Hep Par 1 IHC, Bar = 50 µm.

The immunohistochemical findings show the increased expression of both GPC-3 and Hep Par 1 in the HCC group. The number of positive cells was significantly reduced in the treated groups compared with the HCC group, especially in the group treated with both NSP and sorafenib (Figure 5 and Figure 6). The percentage of expression of both markers is summarized in Supplementary Table S1.

## 4. Discussion

The prognosis for HCC, like other aggressive cancers, is bleak. To date, the Food and Drug Administration has only authorized SOR to treat renal and HCC. However, the benefit is quite small, increasing the median survival time of HCC patients from 7.9 to 10.7 months [27]. Many researchers are now looking into the potential of phytochemicals for treating liver cancer [28]. This study aimed to examine the impact of systemic treatment of sorafenib and *Arthrospira platensis* nanoparticles on DENA-induced HCC in rats. 

A significant decrease in body weight and an increase in liver and relative liver weights were seen in the DENA-treated group compared to the control and other treated groups. Furthermore, the DENA-treated rats showed a significant decrease in food consumption (Data not shown) that was reflected by weight loss and a decline in liver function. As DENA exposure progressed, nodules and tumors developed [18], which may have contributed to the overall increase in liver weight. 

Our findings were in harmony with others [18], who showed a significant loss in body weight and in liver weight in DENA-treated rats. 

On the other hand, other researchers [29] returned the loss of liver weight to the increase of the metabolic activity of the body system in DENA-treated rats. On the contrary, there was increased food consumption and decreased liver nodules of SOR, NSP, or combined SOR/NSP-treated rats. Our results are consistent with those of Elsadek et al. [30], who reported that morphological alterations caused by DENA in a rat model of hepatocellular cancer were almost completely reversed after treatment with SOR, and others [18] reported a decrease in both the average number of visible nodules and the number of malignant nodules as a result of *Arthrospira platensis* treatment. The increase in weight gain and the decrease in absolute and relative liver weight could be attributed to the propensity of SOR, NSP, or combined SOR/NSP-treated rats to sustain and regain their body weights more rapidly. This investigation showed that serum liver activities were significantly increased due to DENA-induced hepatic disruption and the consequent leakage of cytoplasmic and membrane-bound enzymes from the neoplastic cells into circulation. Significant increases in total blood protein and globulin were also seen in the DEN-treated group, although decreases in albumin and the A/G ratio were seen. Liver function, synthetic capacity, and integrity were all boosted in DEN-supplemented rats treated with SOR, NSP, or their combination through preventing enzyme leakage and protecting membrane integrity; SOR and NSP may aid in liver parenchymal and bile ductular cell regeneration in response to the toxic effects of DEN. When DEN is metabolically activated in the liver, reactive oxygen species (ROS) are produced, which are the primary cause of severe oxidative stress. DNA adduct production, lipid and protein damage, and cellular aberrations are only some of the ways in which oxidative stress plays a role in hepatocarcinogenesis [31]. According to our data, DENA treatment dramatically elevated MDA levels while simultaneously decreasing GSH, SOD, and CAT activities. Oral administration of NSP and SOR and their combinations substantially overturned the negative impact caused by DEN. Strong evidence from a prior study indicating that DENA decreased antioxidant enzymes and raised MDA levels supports this finding [18,32]. The elevated MDA level indicates damage to cell membranes and structural changes in the liver in HCC [33,34], which is corroborated by our results.

*Arthrospira platensis* contains numerous bioactive chemicals, which contribute to its antioxidant capabilities [15]. *Arthrospira platensis* protected rat livers from carbon tetrachloride-induced hepatocellular damage [35]. Furthermore, Richard et al. [36] demonstrated the potential for polyunsaturated fatty acids to perform antioxidant functions by scavenging harmful free radicals. Additionally, the total flavonoid and polyphenol levels of *Arthrospira platensis* may be responsible for its hepatoprotective impact in the DEN-induced HCC. Tumor cells are particularly sensitive to polyphenols’ anti-proliferative and anti-angiogenic actions [37].

*Spirulina platensis*, which contains vitamins C and E, may have antioxidant properties. Vitamins A, C, and E are examples of antioxidants. By sequestering peroxyl radicals and keeping GSH levels high, vitamin E may prevent the oxidation of lipid bilayers in cells [38]. Chlorophyll and its derivatives in *Spirulina platensis* mop up free radicals [39].

Regarding tumor biomarkers, the present investigation found that DENA-treated rats had significantly elevated serum AFP and CEA levels. The current investigation results are consistent with my own prior observations. Fathy et al. [40] measured DENA-induced HCC in male Wistar albino rats that showed substantial increases in tumor and liver function indicators. SOR, NSP, or a combination of the two were able to dramatically lower the increased levels of AFP and CEA in DENA-supplemented rats. This results from their anti-tumor effects, most likely caused by decreased tumor production rates. In accordance with the current research results, Liver function enzymes and tumor markers were reduced by SOR in DENA-induced HCC [30]. Following the findings of Assar et al. [18], a histopathological investigation, the tissue architecture of the livers of DEN-treated rats had been significantly altered, suggesting carcinogenicity. In addition, Aly et al. [41] found that the production of reactive oxygen species (ROS) is responsible for the neoplastic alterations in the liver brought on by DEN. HCC-treated rats with NSP showed restoration of hepatic architecture. Differential expression of Glypican-3 (GPC-3) is observed throughout the invasive phase of liver cancer growth, suggesting a role for its expression in the etiology and progression of this disease [42]. This suggested that GPC-3 could serve as a tumor biomarker for HCC. Hep Par-1 aids much in separating HCC from other forms of cancer [43]. This study also demonstrated that hepatocytes expressed Hep Par-1 and GPC-3 at considerably higher levels after DEN injection, lending credence to the hepatocarcinogen’s role in this process. These results are supported by [44]. The hepatocytes expressing Hep Par-1, and GPC-3 were nearly normalized by the SOR, NSP, or their combination.

Cyp19 is a key player in tumorigenesis [45]. Significant up-regulation of Cyp19 was found in rat liver carcinomas, which may be a marker of HCC [46]. Our result revealed that DEN was associated with elevated Cyp19 levels, suggesting tumor initiation. The levels of Cyp19 were decreased in rats given SOR, NSP, or their combination. These results show that NSP affects the initiation of DEN cancer via down-regulating Cyp19.

The p53 gene controls key cellular processes such as proliferation, differentiation, and transformation [47].

Our result revealed a marked decrease (*p* < 0.01) of p53 mRNA observed in the DEN-treated group, which agrees with [48]. In the same way, NSF supplementation showed significant up-regulation of p53. Upon supplementation with *A. awamori*, a significant up-regulation of the level was noticed. This may be due to *Arthrospira platensis* stimulating the production of new p53 protein or other proteins that stabilize p53 [18]. By up-regulating p53 and down-regulating Cyp19, *Arthrospira platensis* maintained a homeostatic balance between cell proliferation and apoptosis, preventing tumor development [18].

Our results revealed that SOR, NSP, or combined NSP /SOR downregulated the inflammatory cytokines with marked improvement in HCC-treated rats with a combined NSP/SOR; these data were in line with [49], where *Arthrospira platensis* reduced the hepatic expression of TNF-, IL-6, iNOS, and TGF-1.

Ki 67 mRNA expression in the liver tissue of HCC rats treated with either SOR, NSP, or a combination of NSP and SOR was significantly downregulated (*p* < 0.05) compared to the DENA group. In addition, the Ki-67 gene expression in the DENA group was significantly increased relative to the other treated groups. The up-regulation of Ki-67 mRNA expression in DENA-induced hepatocarcinogenesis. Ki-67 mRNA overexpression was previously reported in DENA-induced hepatocarcinogenesis. Therefore, these results are in line with those [50]. Surprisingly, Sor, NSP, or combined SOR/NSP treatment decreased Ki-67 gene expression. These findings corroborate prior research showing that SOR suppressed proliferation in HCC mice models by lowering Ki-67 protein expression [51]. A very potent transcriptional activator, FOXO-1, induces the expression of genes involved in cell cycle arrest, apoptosis, DNA repair, and tumor suppression (Yang et al. 2014) (Jiang et al. 2019). The current study found that FOXO-1 gene expression was downregulated in DENA-induced HCC. These results were supported by Xie et al. [52], who reported that evidence of FOXO-1 dysregulation in cancerous tissues existed. In the present study, SOR, NSP, or a combination of NSP and SOR attenuated hepatocarcinogenesis by promoting FOXO-1 mRNA expression. 

PPAR-γ was identified as a tumor suppressor gene and regulates HCC cell apoptosis by modulating the PI3K pathway [53]. In DENA-induced HCC, PPAR-γ-gene expression fell. In agreement with this observation, PPAR-γ-knockout animals were associated with hepatocarcinogenesis. In contrast, PPAR-γ agonists decreased HCC carcinogenesis and metastasis and acted as tumor suppressor genes in the liver [54]. SOR, NSP, or a combination of NSP and SOR up-regulated PPAR-γ-gene expression.

## 5. Conclusions

In DEN-induced hepatocellular carcinoma in rats, the anti-tumor impact of SOR/NSP combination therapy is greater than that of SOR monotherapy. The anti-proliferative, anti-inflammatory, and autophagy signaling pathways that NSP activates are responsible for this action. These effects can be traced back to NSP’s capacity to improve liver histology and function by lowering reactive oxygen species (ROS). NSP’s potential to inhibit sorafenib resistance and stimulate anti-proliferative, anti-inflammatory, and autophagy signaling pathways is responsible for this impact. These results can be traced back to NSP’s ability to restore liver histology and functioning and diminish reactive oxygen species (ROS) thanks to its potent antioxidant action. Additionally, the NSP can reverse the HCC-induced reduction of p53 expression, and SOR/Nps combination therapy reduces SOR resistance in HCC by inhibiting the activation of PPAR- and FOXO-1 and lowering Ki-67 and inflammatory cytokines. These results indicate that SOR/Nps combination therapy is useful for tumor suppression and SOR resistance in HCC.

## Figures and Tables

**Table 1:** Primers for gene expression by RT-PCR.

Gene	Direction	Primer Sequence	Accession Number
CYP19	Sense	CGTCATGTTGCTTCTCATCG	EF110566
	Antisense	TACCGCAGGCTCTCGTTAAT	
p53	Sense	CCCAGGGAGTGCAAAGAGAG	NM_030989.3
	Antisense	TCTCGGAACATCTCGAAGCG	
GAPDH	Sense	TCAAGAAGGTGGTGAAGCAG	NM_017008.4
	Antisense	AGGTGGAAGAATGGGAGTTG	
TNF-α	Sense	GACCCTCACACTCAGATCATCTTCT	NM_012675.3
	Antisense	TTGTCTTTGAGATCCATGCCATT	
iNOS	Sense	TCTTCAAGGACCTACCTCAGGC	S71597.1
	Antisense	GCTAAGGCAAAGCTGCTAGGTC	
TGF B-1	Sense	TCACTTGTTTTGGTGGATGC	NM_021587.2
	Antisense	TTCTGTCTCTCAAGTCCCCC	
PPAR-γ	Sense	TGTGGACCTCTCTGTGATGG	NM_013124.3
	Antisense	CATTGGGTCAGCTCTTGTGA	
FOXO-1	Sense	AGATCTACGAGTGGATGGTG	NM_001191846.3
	Antisense	GGACAGATTGTGGCGAATTC	
Ki-67	Sense	CTTTGCGCCATGCTGAAACT	NM_001271366.1
	Antisense	ATGACGACCTGGACATCGG	

GAPDH, glyceraldehyde-3-phosphate dehydrogenase. CYP19, cytochrome P450. PPAR-γ, Peroxisome proliferator-activated receptor gamma. FOXO-1, Forkhead box protein O1. Ki-67, Proliferation marker protein. TNF-α, tumor necrosisfactor-alpha. iNOS, induced nitric oxide synthase. TGF-1β, transforming growth factor. P53, tumor protein p53.

**Table 2:** Effects of SOR, NSP, and their combination in DENA-induced HCC on body and liver weight.

Liver Relative Weight ^‖^	Liver Weight (g)	Gain Relative Weight *	Body Gain (kg)	Final Weight (kg)	Initial Weight (kg)	Groups
1.98 ^d^	4.97 ± 0.12 ^d^	33.6 ± 2.3 ^a^	0.084 ± 0.01 ^a^	0.25 ± 0.01 ^a^	0.166 ± 0.02 ^a^	Control
4.57 ^a^	6.91 ± 0.22 ^a^	−11.9 ± 1.02 ^e^	−0.018 ± 0.02 ^e^	0.151 ± 0.02 ^d^	0.169 ± 0.01 ^a^	HCC
2.65 ^b^	5.34 ± 0.14 ^b^	15.4 ± 1.15 ^d^	0.031 ± 0.01 ^d^	0.201 ± 0.03 ^c^	0.17 ± 0.011 ^a^	HCC + SOR
2.5 ^b^	5.25 ± 0.16 ^b^	18.5 ± 2.01 ^c^	0.039 ± 0.01 ^c^	0.21 ± 0.01 ^c^	0.171 ± 0.03 ^a^	HCC + NSP
2.30 ^c^	5.12 ± 0.17 ^c^	21.17 ± 2.1 ^b^	0.047 ± 0.02 ^b^	0.222 ± 0.04 ^b^	0.175 ± 0.02 ^a^	HCC + SOR + NSP

Data are presented as the mean ± SE. Mean values with different letters in the same column differ significantly at (*p ≤* 0.05) (*n* = 8). * Body gain relative to the initial weight. **^‖^** Liver relative weight = (liver weight/Final weight).

**Table 3:** Effects of SOR, NSP, and their combination in DENA-induced HCC biochemical parameters.

Groups	ALP (IU/L)	AST (IU/L)	ALT (IU/L)	GGT (IU/L)	Total Protein (g/dl)	Albumin (g/dl)	Globulin (g/dl)
Control	18.1 ± 1.2 ^d^	40.5 ± 1.4 ^d^	23.4 ± 1.2 ^d^	12.22 ± 1.5 ^d^	5.7 ± 0.48 ^b^	3.3 ± 0.11 ^a^	2.2 ± 0.14 ^b^
HCC	25.2 ± 2.1 ^a^	98.3 ± 3.8 ^a^	89.2 ± 2.1 ^a^	27.9 ± 1.2 ^a^	6.2 ± 0.42 ^a^	2.9 ± 0.21 ^b^	3.9 ± 0.25 ^a^
HCC + SOR	21.8 ± 1.3 ^b^	55.2 ± 3.5 ^b^	45.2 ± 1.6 ^b^	19.2 ± 1.3 ^b^	5.1 ± 0.47 ^b^	3 ± 0.14 ^a^	2.1 ± 0.22 ^b^
HCC + NSP	20.1 ± 1.4 ^b^	51.1 ± 2.1 ^b^	40.1 ± 1.5 ^b^	17.6 ± 1.2 ^b^	5.3 ± 0.35 ^b^	3.1 ± 0.25 ^a^	2.2 ± 0.21 ^b^
HCC + SOR + NSP	19.2 ± 1.5 ^c^	47.2 ± 2.5 ^c^	39.1 ± 1.2 ^c^	15.3 ± 1.1 ^c^	5.5 ± 0.74 ^b^	3.2 ± 0.23 ^a^	2.3 ± 0.21 ^b^

Data are presented as the mean ± SE. Mean values with different letters in the same column differ significantly at (*p* ≤ 0.05). (*n* = 8).

**Table 4:** Semi-quantitative analysis of morphometric changes in liver of different groups.

Groups	Cellular Atypia	Macrosteatosis	Fibrosis	Congestion	Mono Nuclear Cells
control	-	-	-	-	-
HCC	+++	+++	+++	+++	+++
HCC+SOR	++	+	+	++	++
HCC+NSP	++	++	+	++	++
HCC+SOR+NSP	+	+	+	+	+

The severity of pathological lesions in different groups of rats was determined according to the percentage of tissue affected: none (-): normal histology with zero involvement of the examined field; mild (+):5–25% of the examined field is involved; moderate (++): 26–50% of the examined field is involved; severe (+++): ≥50% of the examined field is involved. Incidence is the number of rats with lesions per total examined. (*n* = 8).

## Data Availability

Not applicable.

## Supplementary Materials

The following supporting information can be downloaded at: https://www.mdpi.com/article/10.3390/toxics11020107/s1, Figure S1: XRD patterns of Spirulina Platensis NSP; Figure S2: Zeta potential of Spirulina Platensis NSP; Figure S3: Particle size of Spirulina Platensis NSP; Figure S4: Scanning of Spirulina Platensis NSP; Table S1: The percentage of expression of both markers.
