# Supplementary material for: Impending Chemotherapeutic Impact of Arthrospira platensis Nanoparticles and/or Sorafenib against Hepatocellular Carcinoma through Modulation of Antioxidant Status, Tumor Marker Genes, and Anti-Inflammatory Signaling Pathways
Source: Toxics. 2023 Jan 22;11(2):107. doi: 10.3390/toxics11020107 (PMC9964820; doi:10.3390/toxics11020107)
Supplement: Supplementary file 1 [file toxics-11-00107-s001.zip › toxics-2095481-supplementary.pdf]

toxics

Supplementary Materials: Impending Chemotherapeutic Impact of *Arthrospira Platensis* Nanoparticles and/or Sorafenib against Hepatocellular Carcinoma through Modulation of Antioxidant Status, Tumor Marker Genes, and Anti-Inflammatory Signaling Pathways

Heba I. Ghamr

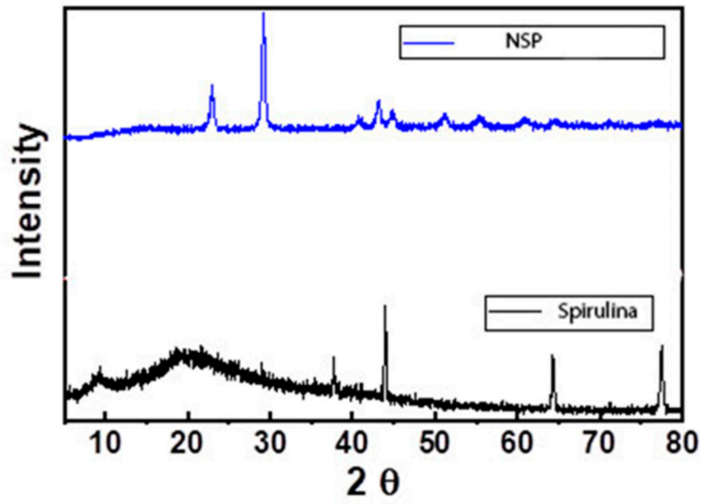

A. XRD patterns of Spirulina Platensis NSP

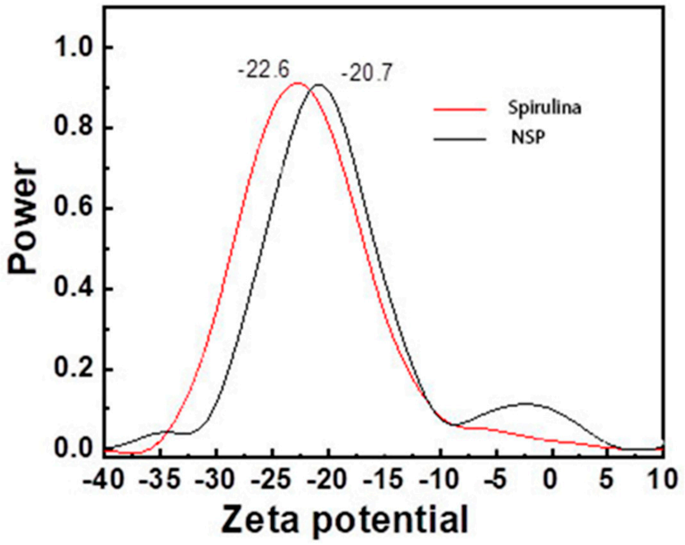

B. Zeta potential of Spirulina Platensis NSP

Figure S1: XRD patterns of Spirulina Platensis NSP;

005

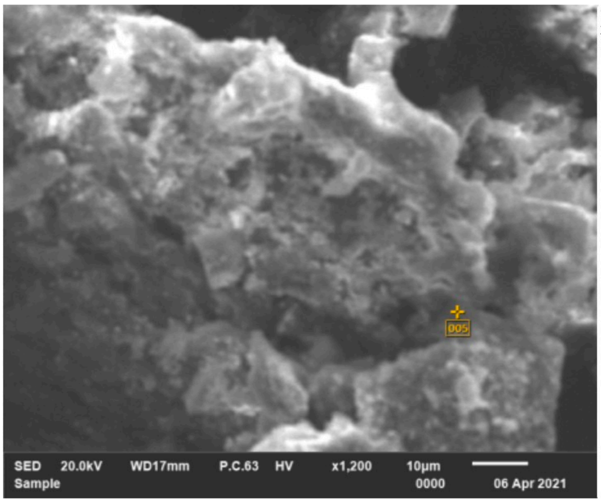


```
Volt          : 20.00 kV
Mag.          : x 1,200
Date          : 2021/04/06
Pixel         : 640 x 480
```

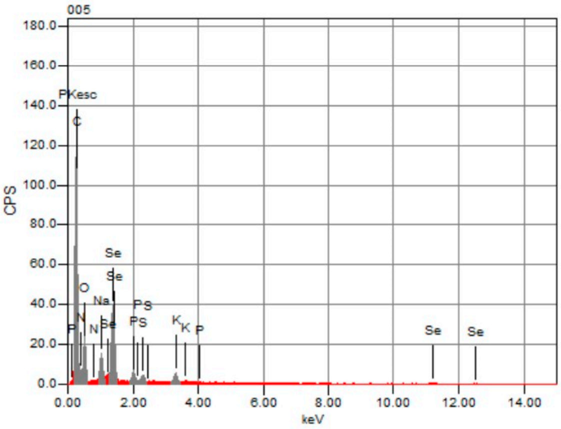


```
Acquisition Condition
Instrument    : IT100LA
Volt          : 20.00 kV
Current       : ---
Process Time  : T3
Live time     : 27.56 sec.
Real Time     : 27.91 sec.
DeadTime      : 2.00 %
Count Rate    : 2285.00 CPS
```

Formula	mass%	Atom%	Sigma	Net	K ratio	Line
C	43.28	52.03	0.03	74805	0.0205412	K
N	26.47	27.29	0.17	7796	0.0290421	K
O	19.45	17.56	0.11	16152	0.0202690	K
Na	1.80	1.13	0.02	13005	0.0066294	K
P	0.45	0.21	0.01	5168	0.0024128	K
S	0.33	0.15	0.01	4098	0.0018321	K
K	0.63	0.23	0.01	6688	0.0038747	K
Se	7.58	1.39	0.07	47746	0.0431695	L
Total	100.00	100.00				

JEOL EDS System JEOL

Figure S2: Zeta potential of Spirulina Platensis NSP;

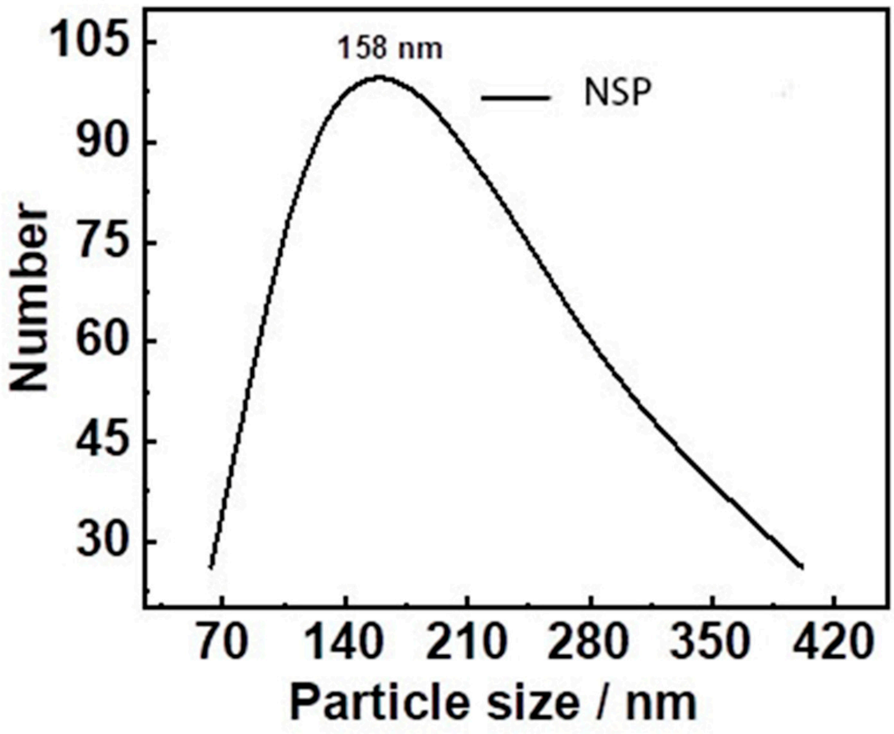

Figure S3: Particle size of Spirulina Platensis NSP;

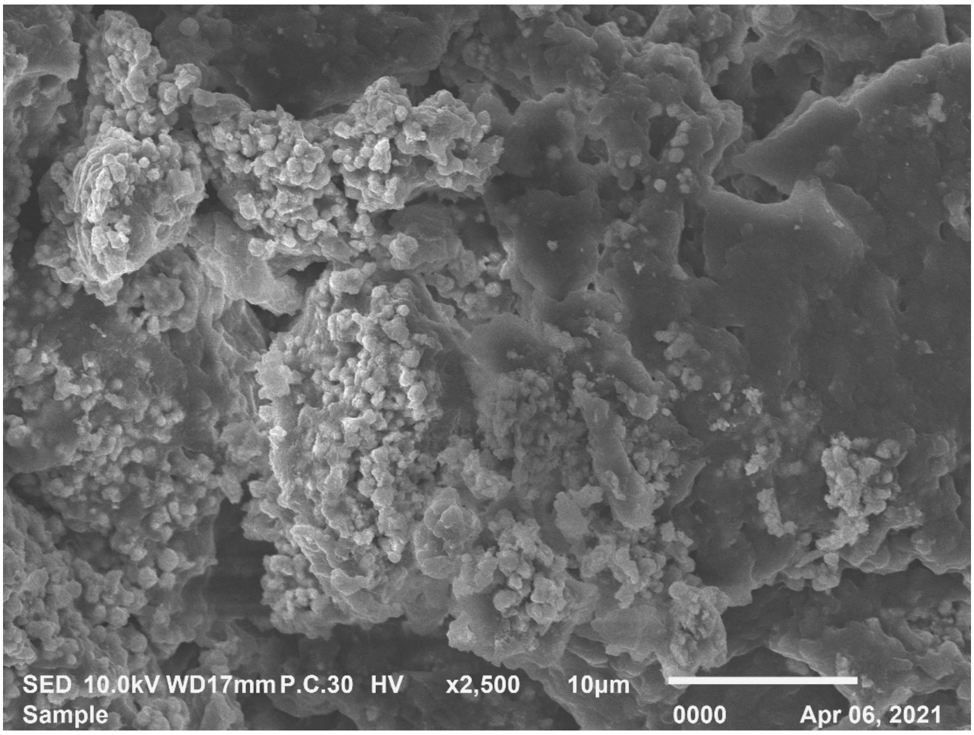

Figure S4: Scanning of Spirulina Platensis NSP;

Table S1. The percentage of expression of both markers.

% of GPC-3 expression/1000 cells	0.0±0.0D	0.4300±0.39a	0.2360±0.02b	0.2820±0.012 b	0.1880±0.017c
% of Hep Par 1 expression/1000 cells	0.06200±0.0 d	0.6140±0.029 a	0.4100±0.013 b	0.3900±0.014 b	0.2620±0.016c

Data are presented as the mean ± SE. Mean values with different letters in the same column differ significantly at ($p \leq 0.05$) ($n = 8$).
